# Cross incompatibility restricting genome exchange and diversification of primary gene pool in plants: challenges and opportunities

**DOI:** 10.1007/s00122-026-05347-x

**Published:** 2026-08-25

**Authors:** Mahendar Thudi, Yogesh Dashrath Naik, Uday Chand Jha, M. S. Sai Reddy, Manish Kumar Pandey, Reyazul Rouf Mir, Muraleedhar S. Aski, Murukarthick Jayakodi, Venugopal Mendu, Naveen Puppala, P. V. Vara Prasad, Somashekhar M. Punnuri, Henry T. Nguyen

**Affiliations:** 1 Centre for Crop Health and School of Agriculture and Environmental Science, University of Southern Queensland, Toowoomba, QLD Australia; 2 Agricultural Science Center, New Mexico State University, Clovis, NM 88101 USA; 3 Crop Improvement Division, Indian Council of Agricultural Research- Indian Institute of Pulses Research, Kanpur, Uttar Pradesh 208024 India; 4 Department of Entomology, Dr. Rajendra Prasad Central Agricultural University, Pusa, Bihar 848125 India; 5 Genomics, Prebreeding & Bioinformatics, International Crops Research Institute for the Semi-Arid Tropics, Hyderabad, Telangana 502324 India; 6 Faculty of Agriculture, Sher-E-Kashmir University of Agricultural Sciences and Technology, Sopore, Jammu and Kashmir 193201 India; 7 Division of Genetics, Indian Council of Agricultural Research-Indian Agricultural Research Institute, Pusa, Delhi 110012 India; 8 Department of Soil and Crop Sciences, Texas A&M University, Texas A&M AgriLife Research and Extension Center, Dallas, TX 75252 USA; 9 Department of Agronomy, Agribusiness & Environmental Sciences, Texas A&M University, Kingsville, TX 78363 USA; 10 Department of Agronomy, Kansas State University, Manhattan, KS 66506 USA; 11 College of Agriculture, Family Sciences and Technology, Fort Valley State University, 1005 State University Dr, Fort Valley, GA 31030 USA; 12 Division of Plant Science & Technology, University of Missouri, Columbia, MO 65211 USA

## Abstract

**Key message:**

Crop wild relatives provide essential genetic diversity for improving crop resilience; however, pre- and post-zygotic barriers often restrict interspecific hybridization. Recent approaches enable their effective use in crop improvement programs.

**Abstract:**

Crop wild relatives (CWR), closely related wild taxa of cultivated crops, offer a wealth of genetic diversity essential for developing traits to help crops adapt to the challenges of biotic and abiotic stresses. This diversity is critical to meet the increasing global demand for food, especially under the looming threat of climate change. However, crossing between individuals from different species often results in maladapted or inviable offspring due to pre- and post-zygotic barriers. Pre-zygotic barriers prevent successful pollen–stigma interactions and pollen tube growth, while post-zygotic barriers include hybrid embryo or endosperm failure and chromosome pairing issues, contributing to cross incompatibility (CI). These barriers restrict genome exchange and hinder the diversification of the primary gene pool in plants. Overcoming these barriers is a significant challenge. This review explores the diverse pre- and post-zygotic barriers in interspecific hybridization, emphasizing genetic factors related to CI. Additionally, this review highlights biotechnological approaches such as somatic hybridization and embryo rescue techniques, which are used to overcome these barriers. Through continued research and innovation, these barriers can be overcome, unlocking the full potential of CWR to address the evolving demands of global agriculture. Recent advances in synthetic biology now offer the possibility to reprogram reproductive barriers, turning CI into a controllable trait for hybrid development.

## Introduction

Improving crop yields remains central to achieving global food security under increasingly complex environmental constraints. Sustainable crop production is challenged by a convergence of biotic and abiotic stresses, further intensified by climate change. These pressures have accelerated the need for innovative breeding strategies. Over the past decades, plant breeding has enabled targeted selection for locally adapted traits, but this has also resulted in a progressive narrowing of the genetic base in cultivated crops (Smykal et al. 2018; Mi et al. 2020). In contrast, crop wild relatives (CWR) retain extensive genetic diversity shaped by long-term recombination, drift, and natural selection (Smykal et al. 2018). Harnessing this diversity through wide hybridization offers a powerful route to introduce novel alleles and enhance adaptive potential (Bohra et al. 2022). However, its application remains constrained by persistent reproductive barriers, including both pre- and post-fertilization mechanisms (Wang and Filatov 2023). Reproductive isolation encompasses mechanisms that restrict gene flow between populations, ultimately driving divergence and speciation (Kulmuni et al. 2020). In flowering plants, this involves multiple coordinated processes, including pollen recognition, pollen tube growth, fertilization, and gamete fusion (Dresselhaus et al. 2016). Morphological traits such as floral structure, color, and phenology also contribute to isolation, particularly when divergence accumulates over time and across geographic scales (Widmer et al. 2009). Genetic factors underpin both pre-zygotic and post-zygotic barriers, with the latter reducing hybrid fitness and the former preventing successful fertilization (Widmer et al. 2009; Wang and Filatov 2023).

Cross incompatibility (CI) is a reproductive barrier that restricts gene flow between genetically divergent parents by preventing successful fertilization or hybrid development, thereby limiting the production of viable hybrids. CI can occur at both intraspecific and interspecific levels. Intraspecific CI occurs between genetically distinct populations, subspecies, or genotypes within the same species, whereas interspecific CI occurs between different species and frequently restricts gene flow between cultivated crops and their CWR. CI represents a major pre-zygotic barrier acting at the pollen–pistil interface, often preventing pollen tube progression to the ovule (Huang et al. 2023). In some cases, post-zygotic effects such as embryo arrest and abnormal development arise from endosperm imbalance, as observed in tomato (Lafon-Placette and Köhler 2016). High sterility in hybrids is frequently associated with pollen abortion or defects in the embryo sac. Notably, CI can also serve adaptive roles, such as preventing unwanted pollen contamination in maize (Maharjan et al. 2022). CI systems have been extensively characterized in crops including maize (*Zea mays*), rice (*Oryza sativa*), *Brassica*, and potato (*Solanum tuberosum*) (Kallugudi et al. 2022; Huang et al. 2023). In maize, the *Ga1* locus comprises multiple linked genes, including pollen-expressed pectin methylesterases (*ZmGa1Ps-m*), a silk-expressed PME (*ZmPME3*), and a pathogenesis-related protein gene (*ZmPRP3*), collectively regulating pollen tube growth and compatibility (Wang et al. 2022a). These insights provide a mechanistic foundation for manipulating CI in breeding programs.

Advances in plant genomics over the past two decades have transformed our ability to dissect complex traits and reproductive barriers. High-throughput sequencing and sequence-based genotyping now enable efficient linkage mapping, genome-wide association studies (GWAS), and genomic selection across large populations (Gangurde et al. 2022). However, CI presents a unique limitation by restricting the development of mapping populations required for genetic mapping and breeding. Furthermore, reliance on a single-reference genome often fails to capture intraspecific diversity, prompting the adoption of pan-genomic approaches to better represent structural and presence–absence variation within species (Khan et al. 2020).

Emerging technologies are beginning to overcome these constraints. Pan-genome-enabled GWAS, single-cell transcriptomics, and AI-driven predictive models provide new avenues to dissect reproductive barriers at high resolution. In parallel, genome editing tools such as CRISPR/Cas systems offer unprecedented precision to modify key genes underlying incompatibility. Together, these approaches enable targeted disruption or bypassing of CI mechanisms, facilitating gene flow from CWR into cultivated gene pools. This review synthesizes current understanding of CI barriers and highlights integrative strategies combining genomics, breeding, and synthetic biology to overcome them. Particular emphasis is placed on identifying divergent genes and haplotypes controlling incompatibility and leveraging these insights to enable efficient introgression. By bridging fundamental reproductive biology with translational genomics, these advances provide a pathway toward expanding crop genetic diversity and enhancing resilience under future climate scenarios.

## The concept of gene pools

The concept of gene pools categorizes crop species and their related counterparts into three distinct gene pools based on genetic similarity and crossbreeding potential (Harlan and de Wet 1971). The primary gene pool includes species readily amenable to crossbreeding with normal chromosomal pairing and segregation. The secondary gene pool comprises closely related species that are crossable with the crop species but exhibit partial reproductive barriers such as reduced hybrid fertility, partial sterility, or irregular chromosome pairing. Despite these constraints, gene introgression from the secondary gene pool is feasible through conventional breeding approaches, often supported by backcrossing, selection, or embryo rescue. This gene pool is particularly valuable because it often harbors alleles for disease resistance, abiotic stress tolerance, and yield-related traits that are absent or rare in cultivated germplasm. The tertiary gene pool features species more distantly related to the primary gene pool, making gene transfer challenging without special measures. While the tertiary gene pool has not yet been extensively utilized in crop improvement, the key challenge lies in harnessing this genetic diversity through wide hybridization to transfer desirable traits, often hampered by CI barriers. Nevertheless, recent advancements in genomics and biotechnology hold promising potential for leveraging the genetic resources of CWR from the tertiary gene pool to enhance the productivity and adaptability of crops. CWR can be a treasure trove for plant breeders (Bohra et al. 2022). These plants have a lot of untapped genetic diversity that could improve crops, mainly because they can grow in all sorts of adverse conditions.

## Insights into cross incompatibility and their barriers

**Fig. 1:**
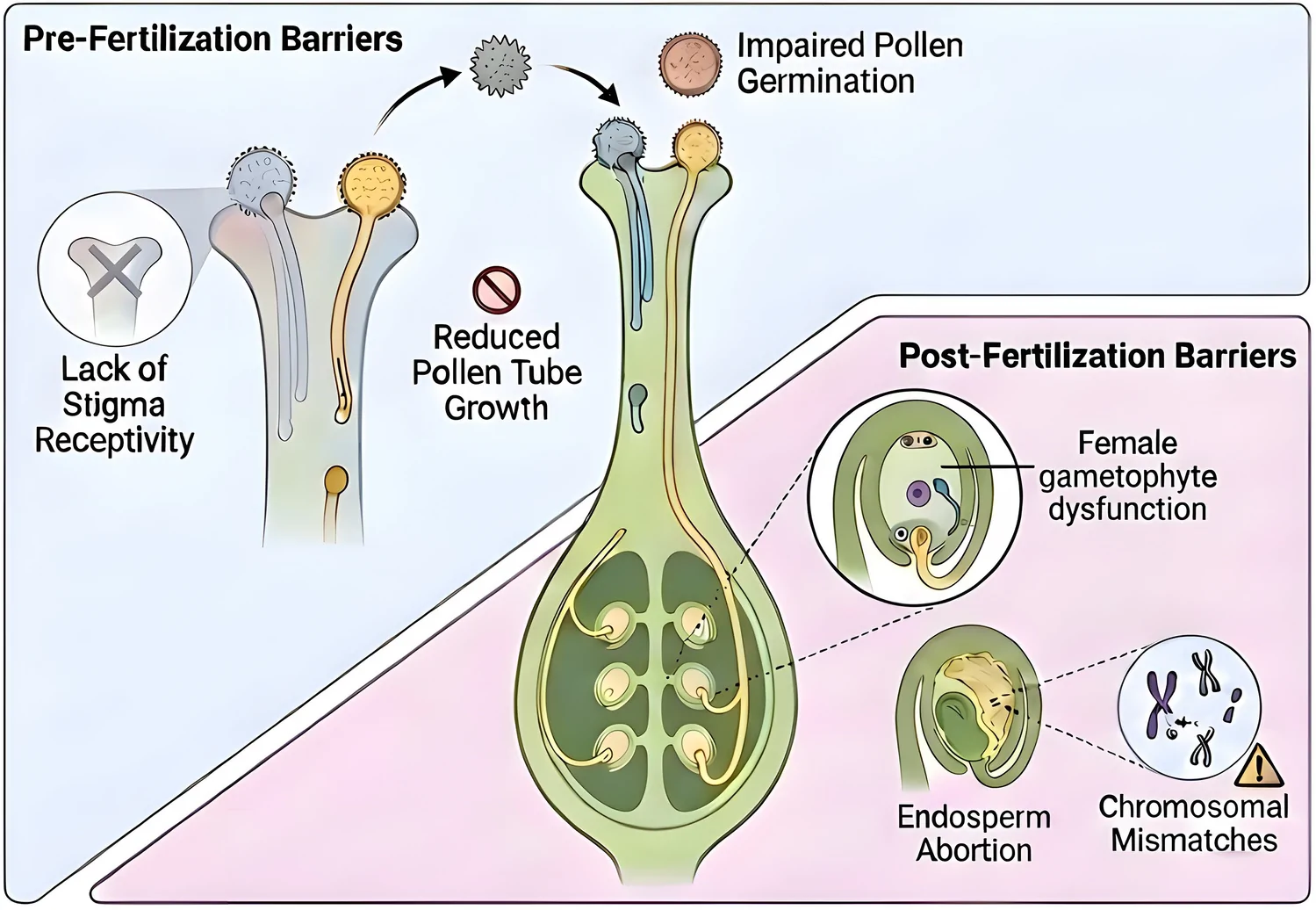
Pre- and post-fertilization barriers underlying plant reproductive incompatibility. Overview of reproductive barriers that prevent successful fertilization in flowering plants. Pre-fertilization barriers (upper panel) include reduced stigma receptivity, failed pollen germination, and inhibited pollen tube growth, preventing fertilization. Post-fertilization barriers (lower panel) occur after fertilization and include female gametophyte dysfunction, endosperm abortion, and chromosomal incompatibility leading to hybrid inviability. Together, these barriers contribute to self- and cross incompatibility and limit gene flow between divergent genotypes and species

Plant breeding involves crossing different species or varieties to create new plant varieties. However, CI can make transferring desirable genes from wild species to cultivated ones difficult. CI occurs when the pollen and pistil of two fertile species fail to form a hybrid zygote. Thomas Fairchild first observed this phenomenon in 1717 when he crossed Carnation × Sweet William. In 1924, Karpechenko created intergeneric hybrids of *Raphanus sativus* × *Brassica oleracea*, and in 1888, Wilhelm Rimpau published his findings on the first fertile allopolyploid Wheat × Rye hybrid (Singh et al. 2021). CI in wide crosses is a common problem in plant breeding. It occurs when two different species or genera are crossed, resulting in sterile offspring or have reduced fertility. This phenomenon is caused by pre- or post-fertilization barriers that prevent the successful fusion of gametes or the development of viable embryos (Fig. 1). CI depends on the genetic distance between the two species or genera. The greater the genetic distance, the more likely it is that CI will occur. Various techniques can overcome CI, including embryo rescue, siliqua culture, ovule culture, ovary culture, and inducing polyploidy. One successful technique involves using a bridge species to overcome incompatibility between two other species. For example, by crossing *L. peruvianum* to *L. chilense* and then crossing the progeny to *L. esculentum*, enabled to overcome the incompatibility between the cultivated tomato and *L. peruvianum* (Poysa 1990). CI can occur at different taxonomic levels depending on the degree of genetic divergence between the parental genotypes. At the intraspecific level (including crosses between subspecies, ecotypes, cultivars, or genetically divergent populations within a species), incompatibility is commonly associated with unilateral incompatibility, pollen–pistil recognition, or hybrid sterility loci, as exemplified by *indica* × *japonica* rice and divergent maize germplasm groups. In contrast, interspecific CI occurs between different species and generally involves stronger reproductive barriers, including pollen rejection, embryo abortion, endosperm failure, and chromosome pairing abnormalities, often requiring embryo rescue, bridge crosses, or chromosome manipulation to obtain viable hybrids. Although both forms involve reproductive barriers, interspecific incompatibility is typically more complex because greater genomic divergence leads to the accumulation of multiple pre- and post-zygotic incompatibility mechanisms.

### Pre-zygotic and post-zygotic barriers

The pre-fertilization or pre-zygotic barriers can be broadly categorized into three types: lack of pollen germination, slow pollen growth, and lack of stigma receptivity (Roda and Hopkins 2019). The first barrier occurs due to the failure of pollen to germinate on the stigmatic surface. For example, a cross between *Capsicum frutescens* and *C. chinense* is impossible because *C. chinense* pollen does not germinate on the stigma of *C. frutescens*. Similarly, crossing *L. peruvianum* and *L. esculentum* is difficult because the pollen tube growth of *L. esculentum* is inhibited in the style of *L. peruvianum*. The second type of barrier is caused by the slow growth of pollen, which reduces fertilization success. Supporting these observations, Li et al. (2025) identified significant pre-fertilization barriers in reciprocal crosses between Lagerstroemia indica and *Lythrum salicaria*, where abnormal pollen tube growth led to reduced fertilization success and increased fruit abscission. The third type of barrier is related to a lack of stigma receptivity, as seen in crosses between *Prunus dulcis* and *P. persica* during pollen shedding from *P. dulcis*.

Pollen–pistil interactions comprise a sequence of molecular recognition events occurring between pollination and fertilization, determining the acceptance of compatible pollen and the rejection of self-incompatible or interspecific pollen (Wang and Filatov 2023). Consequently, pollen–pistil interactions play a pivotal role in reproductive isolation and angiosperm speciation. Following successful pollen germination and tube growth, post-fertilization (post-syngamic) barriers may further restrict hybrid formation. These barriers generally occur through three mechanisms: failure of gametic fusion, abnormal endosperm development, and chromosomal incompatibilities. For example, in maize, a cross between *Z. mays* and *Tripsacum dactyloides* is impossible because the male gametes of *T. dactyloides* are unable to fertilize the egg cells of *Z. mays*. The second type of post-fertilization barrier is the failure of normal endosperm formation, which leads to embryo abortion or the development of sterile offspring (Walter et al. 2019). The third barrier is restricted chromosome pairing, leading to abnormal chromosome pairing and segregation during meiosis, which produces inviable offspring.

## Intergeneric crosses/distant hybridization

Intergeneric crosses, also known as distant hybridization, refer to the crossing of two different genera or higher-ranking taxa, which can result in the breaking of species boundaries, increasing genetic diversity, and merging the biological traits of existing species (Liu et al. 2005). In agricultural contexts, intergeneric crosses are often used to transfer desirable genes from secondary and tertiary gene pools to the cultivated “species” genome background (primary gene pool). Plant and animal taxonomists typically use: The Taxonomic Species Concept (TSC) and the Biological Species Concept (BSC). The TSC is based on morphological phenotypes, while the BSC is based on breeding relationships (Grant 2003). The Phylogeny Species Concept (PSC) by Cracraft (1982) is a third concept that is an alternative to the BSC and implies knowledge of whether or not regular interbreeding occurs between populations. Unlike the BSC, the PSC is less restrictive, and breeding between members of different species does not pose a problem. Higher plants are commonly classified using the TSC, which involves comparing the morphological traits of individual plants with holotypes or other reference specimens when holotypes are unavailable (McNeill et al. 2012). In some cases, molecular data is also used alongside morphology for classification. The TSC defines species boundaries solely based on phenotypic traits, assuming species are unchanging over time. In contrast, the BSC defines species as groups of naturally interbreeding populations reproductively isolated from others. Although the BSC excludes asexual reproduction and presents challenges in determining species boundaries, it more accurately reflects natural processes. The BSC is also advantageous for developing breeding strategies to enhance genetic diversity in crops by introducing beneficial traits from their CWR. In higher plants, reproductive isolation arises due to various breeding barriers, including external factors (e.g., geographical, temporal, mechanical) and internal barriers. Internal barriers, expressed within plant tissues, can be pre-zygotic or post-zygotic, depending on whether they occur before or after fertilization. Two key pre-zygotic barriers at the pollen–pistil level self-incompatibility (SI) and CI—prevent pollen tubes from reaching the eggs. SI promotes outcrossing, while CI limits gene flow, potentially leading to or reinforcing speciation (de Nettancourt 2001).

## The factor responsible for cross incompatibility in intergeneric crosses

CI is a type of pre-zygotic hybridization barrier that occurs at the pollen–pistil interface, where the pollen from one species cannot fertilize the ovules of another species. The pollen tube may fail to grow, or it may grow, but the fertilization process is unsuccessful. CI may be due to the pistil or pollen’s genetic, molecular or biochemical differences. In many cases, CI is genetically determined by a single locus, the S-locus, or the SI locus. A comprehensive list of genes and QTLs associated with CI across various crops is provided in Table 1. Historically, the genetic basis of CI was first investigated through linkage mapping, leading to the identification of numerous quantitative trait loci (QTLs) associated with pollen fertility, hybrid sterility, and reproductive barriers. Advances in high-resolution mapping, whole-genome sequencing, and functional genomics have subsequently enabled the cloning and molecular characterization of several major CI genes underlying these loci. Accordingly, Table 1 distinguishes historically identified QTLs from functionally validated genes, illustrating the progression from genetic mapping to mechanistic understanding of CI. The S-locus encodes for a glycoprotein in the pistil’s style that acts as a receptor for the pollen tube (Ma et al. 2016). If the pollen carries the same S-allele as the pistil, the pollen tube growth is inhibited, leading to CI. CI can also be due to differences in the surface chemistry of the pollen and pistil, leading to the recognition and rejection of foreign pollen. For example, intergeneric crosses between members of the Brassicaceae family (e.g., *B. napus* and* R. sativus*) are often unsuccessful due to CI. *Brassica napus* has a SI system that recognizes and rejects foreign pollen. On the other hand, *R. sativus* does not have a SI system, making it incompatible with *B. napus*. Recent work reveals that mechanical constraints within the pollen–pistil interface can act as decisive pre-zygotic barriers in CI. The pollen tube must navigate complex structures—stigmatic papillae, transmitting tracts, and style tissues—and any mismatch in cell wall rigidity or structural guidance can halt growth. Notably, asymmetric PMEI-regulated callose deposition in *Linum* and other species has been shown to physically block interspecies pollen germination, emphasizing a mechanical “lock-and-key” model of reproductive isolation. These biomechanical facets, though often overlooked, are evolutionarily stable and heritable, suggesting they play a critical role in compatibility decisions. Simultaneously, epigenetic regulation—particularly through small RNAs and DNA methylation—has emerged as a key factor in endosperm-based post-zygotic barriers. In wide and interploidy crosses, small RNA imbalances disrupt parental genome dosage and imprinting control, leading to seed abortion—a phenomenon recently linked to maternal “sirenRNAs” in *Arabidopsis* and other species. Moreover, manipulations of DNA methylation (e.g., via 5-azacytidine) have been shown to relieve hybrid seed lethality in *Arabidopsis* interploidy barriers. These rearrangements highlight how epigenomic context shapes compatibility independently of DNA sequence, offering potential avenues for intervention through demethylation or epigenome editing. Recent studies integrating comparative genomics, transcriptomics, and epigenomics have further demonstrated that CI is governed by complex regulatory networks involving structural variation, gene expression divergence, and epigenetic modulation rather than single compatibility loci alone (Wang and Filatov 2023; Zhang et al. 2026). Together, these insights illustrate that CI cannot be fully understood through genetics alone; instead, a multilayered model combining biomechanics and epigenetics is essential. Going forward, integrating biophysical measurements of stylar rigidity, alongside single-cell epigenomics or live small RNA mapping, will be key to unveiling how mechanotransduction and epigenetic states interplay to regulate cross-species fertilization success.

**Table 1:** List of gene/QTL conferring cross incompatibility in various crops

Crop	Cross incompatibility gene/locus/QTL	Details	References
Maize	*Teosinte crossing barrier*1 (*Tcb*1)	*Tcb*1 linked to *gametophyte-*1 locus leads to non-receptivity to the pollen of other maize varieties	Evans and Kermicle (2001)
	*Ga*2	Promotes rejection of the pollen grain by *Ga*2 silks	Kermicle and Evans (2010)
	*ga*1*and ZmPme*3	Causes unilateral cross incompatibility	Lauter et al. (2017)
	*Gametophyte factor*1 (*Ga*1)	Pectin methylesterase gene at the *Ga*1 locus confers the male function in the maize unilateral cross incompatibility	Zhang et al. (2018)
	*Tcb1*	Pectin methylesterase homolog *qSF*5.1 pollen rejection	Lu et al. (2019); Zhang et al. (2023)
	*Ga*2 locus	Male determinant *ZmGa*2*P* and female determinant *ZmGa*2*F* both encoding putative pectin methylesterases control pollen–silk compatibility in different maize genotypes	Chen et al. (2022)
	*ZmGa1P*	Seven linked genes underlie the locus, including pollen-expressed *ZmGa1Ps-m*, silk-expressed *ZmPME*3, and *ZmPRP*3*. ZmPME*3 enables silk to reject incompatible pollen, while *ZmPRP*3 promotes its growth, overcoming *ZmPME*3*’s* blocking effect	Wang et al. (2022a)
	*ZmGa1P*	The *ZmGa1P* contains one silk-expressed pectin methylesterase gene (*ZmGa1F*) and eight pollen-expressed PMEs (*ZmGa1P* and *ZmGa1PL1-7*)	Zhang et al. (2023)
Rice	*Cif* and *cim*	When female plant with *Cif* was crossed with a male plant with *cim* it led to failure of early endosperm development	Matsubara et al. (2003)
	*qSF*5.1*, qSF*6.1 *qSF*6.2*, qSF*3.1*, qSF7, qSF*12.2 *and qSF*9.1	Causing hybrid sterility in the progenies developed from Ilpumbyeo (*japonica*) and Dasanbyeo (*indica*)	Reflinur et al. (2012)
	*DOPPELGANGER*1 (*DPL*1) and *DOPPELGANGER*2 (*DPL*2)	*DPL*s hybrid incompatibility genes encode highly conserved, plant-specific small proteins in mature anther. Pollen possessing defective *DPL* genes causes a defect in pollen germination	Mizuta et al. (2010)
	*S*5* locus*	Inhibits indica/japonica-derived crosses	Chen et al. (2008); Ji et al. (2010, 2012)
	*qS*12	Causes hybrid male sterility in the progenies developed from *indica* × *japonica* cross	Zhang et al. (2011)
	*Hwi*1	Hybrid weakness was obtained in the progenies derived from *Oryza sativa* × *O. rufipogon* cross due to *Hwi*1 gene in wild species of rice	Chen et al. (2013)
	*Hwi*1 (*LOC_Os*11*g*07230 and *LOC_Os*11*g*07240)	Causes hybrid weakness in rice	Chen et al. (2014)
	*Sa*	Promotes loss of fertility in the progenies obtained from cultivated rice weedy rice cross	Craig et al. (2014)
	*Sa, Sb* and *Sc*	Promotes loss of fertility in the progenies obtained from *Oryza sativa ssp. indica* × *japonica*	Wu et al. (2015)
	*Sc*	*Sc* locus promotes *japonica–indica* hybrid male sterility. The *japonica* allele, *Sc-j*, possesses a pollen-essential gene encoding a DUF1618-domain protein. The *indica* allele, *Sc-i*, contains two or three tandem-duplicated ~ 28-kb segments	Shen et al. (2017)
	*Sb* locus	Causes pollen sterility in autotetraploid rice hybrids	Wu et al. (2019)
	*qSS12*, *qSS8*, *qSS11*, *ePS6*-1, and *ePS6*-2	These QTLs promotes spikelet fertility in the progenies derived from wide crosses in rice	Lee et al. (2021)
	*S5* is a major-effect locus with *ORF*3*, ORF*4, and *ORF*5 genes	S5 causes hybrid sterility in progenies derived from crossing indica rice and japonica rice	Rao et al. (2021)
	*qHMS*4	It causes hybrid sterility in the progenies derived from cross between *japonica* rice and *O. glaberrima*	Wang et al. (2022b)
Barley	Single-dominant gene	Promotes partial incompatibility in *Hordeum vulgare L. cv. Vada* × *H. bulbosum L*	Pickering (1983)
Wheat	*Kr*1	Presence of the dominant allele *Kr*1 promotes inhibition of wheat/rye crossability	Riley and Chapman (1967)
	*Kr* genes (*Kr*1 and *Kr*2)	*Kr* genes promote prevention of interspecific crossing by inhibiting pollen tube growth	Bertin et al. (2009)
	*Kr*1 and *SKr*	Both affect the crossability of wheat and rye	Alfares et al. (2009)
*Brassica oleracea*	S-locus glycoprotein (*SLG*) gene and S-locus receptor kinase (*SLR*) gene	*SLG* and *SLR* genes cause SI response by recognizing the self-pollen	Nasrallah et al. (1987); Stein et al. (1991)
	*SCR, SRK, THL*1*, ARC*1, and* MLKP*	These genes promote self-compatibility in *B. napus*, *B. oleracea* and* B. rapa*	Gaude et al. (1993); Isokawa et al. (2010); Murase et al. (2004); Hatakeyama et al. (2010)
	*qSC*7.2*, qSC*9.1 *and* qSC5.1	These QTLs promote self-compatibility in* B. oleracea*	Xiao et al. (2019)
Tomato	S-RNase allele at *SI* locus	30 S-RNase allele sequences in *Solanum chilense*	Igic et al. (2007)
	*HT-A* and *HT-B*	Promotes unilateral incompatibility and self-incompatibility	Covey et al. (2010)
	*HT*-*A* or *HT*-*B*	*HT-A* or *HT-B* gene in the pistil promotes rejection of pollen from all four red-fruited species	Tovar‐Mendez et al. (2014)
	*ui*1.1	*ui*1.1 encoding S-locus F-box protein helps in determining pollen specificity	Li and Chetelat (2015)
	*ui*6.1	Regulates unilateral incompatibility in interspecific Solanum hybrids	Li et al. (2010)

## Insect pollination in intergeneric crosses

Insect pollination can facilitate overcoming CI barriers in intergeneric crosses by breaking pre-zygotic hybridization obstacles. Insects such as bees, butterflies, moths, flies, and beetles, along with other animals, pollinate approximately 80% of all flowering plants, including critical crop species (Ollerton et al. 2011). These pollinators help transfer pollen between plant species, thereby increasing the chances of fertilization and seed formation, which can help break CI barriers caused by pollen–stigma incompatibility, pollen tube failure, or differences in floral structures (de Nettancourt 2001). Insects are also vital for pollinating various crop species, significantly increasing yield and seed production. For example, bees are the most effective pollinators of sunflowers (*Helianthus annuus*), blackberry (*Rubus* spp.), blueberry (*Vaccinium* spp.), and pumpkins (*Cucurbita* spp.), contributing to increased yields in these crops (Garibaldi et al. 2014; Delaplane and Mayer 2000). Passionflowers (*Passiflora* spp.) and lavender (*Lavandula* spp.) also benefit from insect pollination, particularly from bees, which enhance fruit set, seed production, and essential oil quality (Fay et al. 2009; Ballantyne et al. 2015). In the context of intergeneric hybrids, insects such as bees can transfer pollen across genera, facilitating the creation of hybrids like *plumcot* (a hybrid of *Prunus* and *Armeniaca*), *Tangelo* (a hybrid of *Citrus paradisi* and *C. tangerina*), and *Boysenberry* (a hybrid of *Rubus ursinus* and *Rubus idaeus*). For some hybrids like *Triticosecale*, artificial pollination using bees, such as *Osmia rufa*, enhances cross-pollination.

Insect pollination is increasingly recognized not just for enhancing yield but also as a deliberate strategy to overcome CI in plant breeding. Recent evidence shows that managed insect-mediated pollen transfer, from both bees and non-bee insects, can circumvent temporal, morphological, and stylar reproductive barriers that often thwart interspecific and intergeneric crosses. Kamiński et al. (2025) reported that using insect pollinators in *B. rapa* × *Sinapis alba* hybridizations enabled the successful formation of 48 F₁ hybrids, an outcome previously limited by unilateral incompatibility. In *Camellia oleifera*, artificial pollination designed to simulate insect behavior effectively bridged flowering-time mismatches, overcoming pre-zygotic style-based barriers in otherwise incompatible crosses (Gong et al. 2023). Meanwhile, Jing et al. (2021) demonstrated that honeybee pollination significantly outperformed hand pollination in *Trifolium pratense*, yielding higher seed set despite SI. Beyond bees, a comprehensive survey of 39 field studies found that non-bee insects, including flies, beetles, and butterflies, account for approximately 39% of pollinator visits to crops, substantially contributing to fruit set and genetic exchange (Rader et al. 2016). In the realm of plant breeding, precision pollination, the intentional use of data and technology to guide pollinator behavior, emerges as a promising strategy to overcome CI barriers. A pilot study by Ratnayake et al. (2022) deployed computer vision systems across polytunnels to track pollinators, quantify visitation rates, and predict hybridization outcomes in real time, effectively identifying pollinators most adept at transferring interspecific pollen (Ratnayake et al. 2022). Simultaneously, advances in robotic pollinators, such as Bramble Bee for greenhouse crops and autonomous pollination systems for apple orchards, have demonstrated over 90% flower detection accuracy and up to 84% targeted pollination success, showcasing their potential to deliver pollen precisely across compatibility boundaries (Ohi et al. 2018; Sapkota et al. 2023). These technological innovations point toward a future where breeding programs use smart systems to coordinate pollinator presence with flowering windows, ensuring efficient pollen transfer even amid stylar mismatches or temporal incompatibilities. By integrating real-time behavioral data, artificial intelligence, and precision machinery, this approach could redefine hybridization workflows, shifting from passive pollination to engineered ecological processes that actively dismantle reproductive barriers and accelerate the development of novel crop varieties adapted to a changing climate.

## Effect of ploidy on cross incompatibility

Polyploidy is when an organism possesses more than two sets of chromosomes. It is a common mechanism of adaptation and speciation in plants and is estimated to be present in 47–70% of angiosperm species (Pele et al. 2018). The polyploidization process has contributed significantly to the evolution and speciation of angiosperms. These processes can impact various aspects of plants, including their genome, morphology, physiology, and reproductive system (Vaskova and Kolarcik 2019). In flowering plants, the “triploid block” refers to the phenomenon that hinders interploidy hybridizations, leading to a failure in endosperm development, embryogenesis arrest, and seed collapse (Kohler et al. 2021). Polyploidization has been associated with changes in ornamental traits in plants as it increases genetic redundancy, allowing for extensive genetic rearrangements leading to the stable expression of novel traits (Baker et al. 2017). Interploidy crosses between diploid and triploid cultivars of *H. macrophylla* resulted in genetically unstable progeny that showed poor growth and ornamental quality compared to their parent cultivars. Viable seed from controlled interploidy crosses was difficult to obtain, and survival rates were low (Alexander 2020). The same study also reported pollen tube length after pollination was significantly influenced. These findings suggest that both the plant’s genetic makeup and the flower’s physical characteristics can significantly impact the growth and development of pollen tubes. However, polyploidization has been used to develop sterile cultivars, enhance stress tolerance, and improve ornamental attributes in plants, making it an attractive tool for plant breeding and agriculture (Alix et al. 2017). Coupling pollinator behavior tracking with molecular pollen tagging has emerged as a tool to enhance hybridization success and quantify intergeneric pollen flow under field conditions.

## Role of transposable elements in functional evolution of cross incompatibility

Transposable elements (TEs) are sequences of DNA that can move or transpose within a genome. TEs have been shown to have a wide range of effects on the host genome, including altering gene expression, disrupting gene function, and causing chromosomal rearrangements (Wei and Cao 2016). Hybridization between divergent genomes can disrupt the balance between TEs and host silencing systems, leading to TE reactivation, genome instability, and the formation of structural variants (SVs). These TE-driven changes may reduce gene flow and promote reproductive isolation, while in some cases SVs such as inversions can maintain adaptive allele combinations under ongoing gene exchange (Serrato-Capuchina and Matute 2018; Lammers et al. 2026; Wen et al. 2026). Thus, TE reactivation represents a key mechanistic link between hybridization, chromosomal reorganization, and the evolution of reproductive barriers (Lammers et al. 2026). In citrus, a 786-bp MITE insertion in the promoter of *FhiS2-RNase* suppresses gene expression and causes breakdown of self-incompatibility, while its removal restores the self-incompatibility phenotype, confirming a direct regulatory role of TEs in reproductive compatibility shifts (Hu et al. 2023). It was observed that TEs are commonly linked with hybrid defects that could hinder species fusion. Still, their role in other forms of gene flow barriers, apart from post-zygotic isolation, remains largely unclear. TEs are increasingly recognized as important contributors to plant evolution because they drive genome size changes and generate new coding and regulatory sequences. In conjunction with polyploidization events, TEs are believed to be major drivers of plant evolution (Vicient and Casacuberta 2017).

## Conventional efforts to break the CI barriers

Transferring desired traits from wild crops to local cultivars is important in crop breeding to improve the genetic diversity and enhance the adaptation of the cultivated crop to various environmental stresses, such as disease, pests, and abiotic factors like drought and heat (Renzi et al. 2022). Wild crops often possess unique traits absent in the local cultivars, such as resistance to diseases, pests, and environmental stresses. However, there are several barriers to transferring desirable traits from wild crops to local cultivars. One of the main barriers is genetic incompatibility, which can prevent the successful hybridization between the wild and cultivated plants (Laugerotte et al. 2022). In papaya, an experiment on pollen germination with and without sucrose treatment revealed that sucrose significantly enhanced pollen germination and pollen tube growth (Dinesh et al. 2007). This finding can have significant implications for hybridization and breeding programs to improve crop yields and quality.

Additionally, the inter-varietal hybridization between the varieties Surya and Pusa Dwarf resulted in a high fruit set percentage of 91.7% with 300 viable seeds per fruit (Dinesh et al. 2007). In addition to sugar, salt solution treatment is an effective technique in overcoming pre-fertilization barriers during Asiatic and Oriental lily crossbreeding (Fu et al. 2021). To improve seed set during crossbreeding of plants, studies have found that treating the stigma with specific plant hormones can be effective. Combining the auxin 2,4-dichlorophenoxyacetic acid and cytokinin led to a significantly higher pollen tube length to total style length ratio and better seed set (Dhooghe et al. 2021). The pre-fertilization barriers in Chrysanthemum were overcome using special pollination techniques, including mentor pollen, delayed pollination, and gibberellic acid treatment. These techniques were effective in breaking pre-fertilization barriers and increasing seed set in wide hybridization (Sun et al. 2011). Chemically induced epi-mutagenesis was also used to bypass reproductive barriers in *Arabidopsis* plants, treated with the DNA methyltransferase inhibitor 5-Azacytidine during seed germination and early growth, and facilitates hybridization between different species (Huc et al. 2022). Because the nature of CI varies considerably among crop species, the underlying reproductive barriers and the most effective mitigation strategies are also crop specific. Table 2 summarizes the major factors limiting successful hybridization across representative crops together with the conventional and genomic approaches currently employed to overcome these barriers. This comparison highlights that no single strategy is universally applicable and emphasizes the importance of tailoring breeding interventions according to the biological basis of incompatibility.

**Table 2:** Crop-specific cross incompatibility barriers, major limiting factors, and targeted strategies to overcome reproductive barriers in plant breeding

Crop	Major CI barrier/gene(s)	Primary limiting factor	Targeted strategy to overcome CI
Maize	*Ga*1, *Ga*2, *Tcb*1, *ZmPME*3	Pollen rejection, unilateral incompatibility	Compatible alleles, CRISPR editing, marker-assisted breeding
Rice	*S*5, *Sa*, *Sc*, *DPL*1*/DPL*2, *Hwi*1	Hybrid sterility, embryo sac abortion, pollen sterility	Wide hybridization, bridge lines, genome editing, embryo rescue
Wheat	*Kr*1, *Kr*2, *SKr*	Pollen tube inhibition, poor crossability	*SKr* alleles, embryo rescue, chromosome manipulation
Barley	Dominant incompatibility locus	Partial incompatibility with *Hordeum bulbosum*	Embryo rescue, chromosome elimination techniques
Brassica	*SRK*, *SCR/SP*11, *SLG*, *ARC*1	Self- and CI, pollen recognition	CRISPR-mediated gene editing, mentor pollen, bud pollination
Tomato	*S-RNase*, *HT-A*, *HT-B*, *ui*1.1, *ui*6.1	Unilateral incompatibility, pollen rejection	Bridge species, embryo rescue, genome editing
Potato	*S-RNase*, HT genes	Pollen rejection, self-incompatibility	CRISPR-mediated knockout of SI genes, bridge crosses
Soybean	Chromosome divergence, embryo abortion	Hybrid sterility, poor embryo development	Embryo rescue, bridge hybridization, chromosome doubling
Peanut	Genome divergence between wild and cultivated species	Embryo abortion, hybrid sterility	Synthetic amphidiploids, embryo rescue, chromosome doubling
Pigeonpea	Interspecific reproductive barriers	Embryo abortion, reduced fertility	Embryo rescue, backcross introgression, pre-breeding
Chickpea	Crossability barriers with wild *Cicer* species	Hybrid sterility, poor seed set	Embryo rescue, bridge crosses, marker-assisted introgression

## Biotechnological and genomic approaches to break the CI barrier

**Fig. 2:**
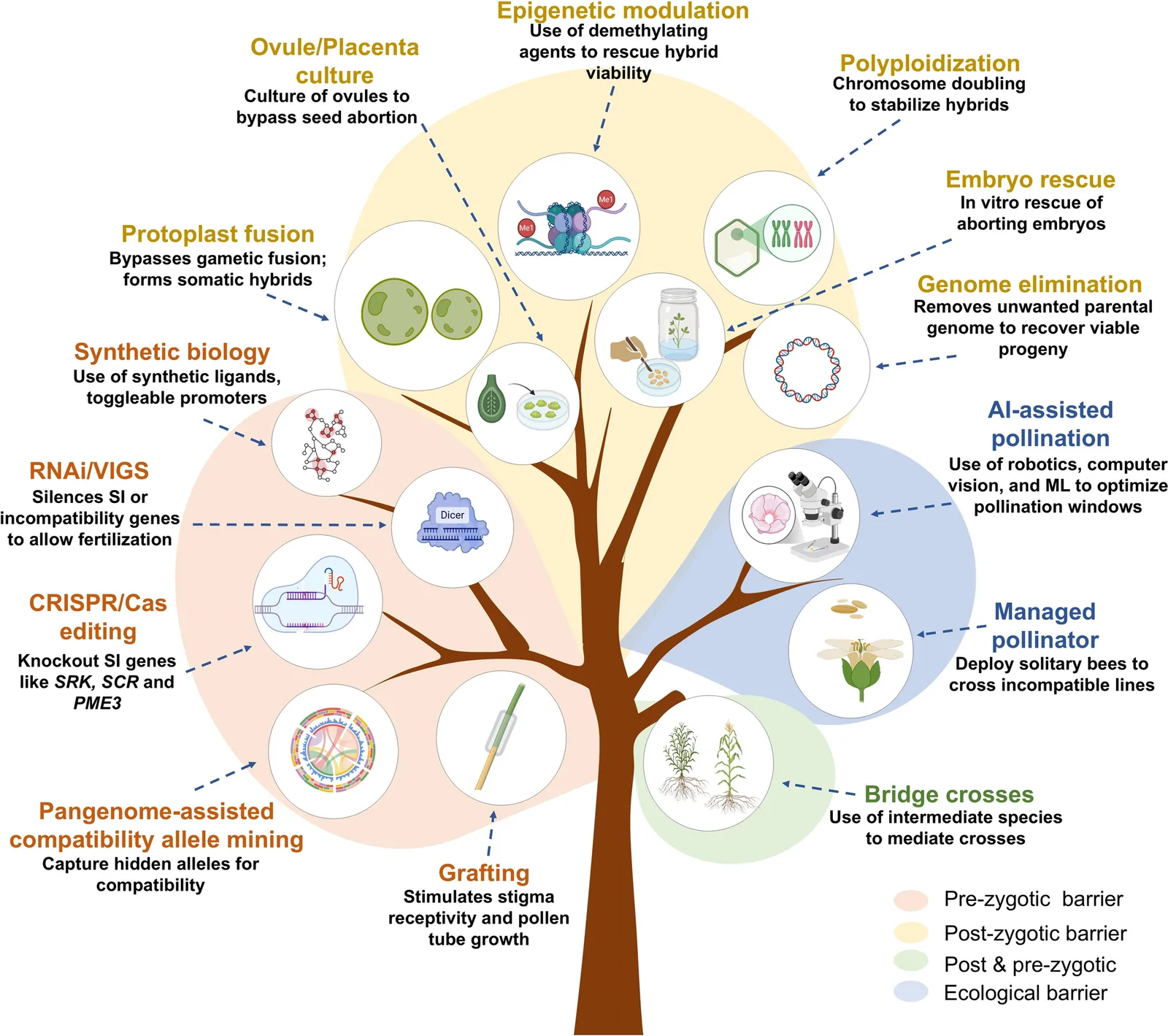
Comparative overview of classical versus modern strategies to overcome cross incompatibility (CI) in plants. Classical methods primarily rely on tissue culture and empirical breeding practices to bypass developmental failures, while modern approaches employ genome editing, synthetic biology, and AI-assisted ecological optimization. Together, these methods target different barriers: genetic, mechanical, and epigenetic to enable hybridization across taxonomic boundaries

Combining conventional biotechnological approaches with modern genomic tools provides a multilayered strategy to overcome CI and improve introgression efficiency in crops (Fig. 2). CI has long limited the transfer of beneficial alleles from CWR, constraining genetic improvement. Among classical approaches, embryo rescue remains a widely used technique, enabling the recovery of hybrid embryos that would otherwise abort. By excising immature embryos and culturing them in vitro, viable plants can be regenerated (Rogo et al. 2023). Similarly, somatic hybridization through protoplast fusion allows the combination of genomes from different species or genera, bypassing sexual incompatibility barriers. These approaches, complemented by genetic engineering and genome editing, provide a robust framework to overcome CI and expand genetic diversity.

Soybean provides an important example of crop-specific reproductive barriers during wide hybridization. Although cultivated soybean (*Glycine max*) is largely compatible with its annual wild progenitor (*G. soja*), facilitating the introgression of useful alleles for stress tolerance and agronomic improvement, reproductive barriers become progressively stronger when crosses involve perennial *Glycine* species. These distant crosses frequently suffer from pollen–pistil incompatibility, embryo abortion, hybrid sterility, chromosome pairing abnormalities, and reduced fertility, thereby limiting gene transfer from valuable wild germplasm. Consequently, embryo rescue, ovule culture, bridge hybridization, and chromosome doubling have been widely employed to recover viable hybrids and facilitate introgression breeding. Recent advances in soybean genomics, including whole-genome sequencing, high-density molecular markers, and pan-genome resources, have further improved the identification and deployment of favorable alleles from CWR while reducing linkage drag. Approximately 89% of wild soybean populations exhibit medium to high sexual compatibility with cultivated soybean following artificial hybridization (Hu et al. 2022), demonstrating the considerable potential of wild germplasm for soybean improvement. Similarly, embryo rescue enabled the development of advanced backcross populations in *Cajanus*, soybean introgression from wild *Glycine* species, and disease-resistant peanut lines derived from CWR (Sharma et al. 2020). These examples highlight the continued relevance of classical approaches in overcoming CI.

Genomic interventions provide a complementary and increasingly powerful strategy to dissect and overcome CI. Identifying genes underlying reproductive barriers is central to enabling targeted manipulation. In rice, hybrid sterility between indica and japonica subspecies is governed by loci such as *Sa, S5,* and *DPL1/DPL2*, which affect gamete viability and embryo sac fertility (Chen et al. 2008; Mizuta et al. 2010). The *S5* locus encodes an aspartic protease that disrupts embryo sac development, while *Sa* regulates male sterility. Additional loci such as *Hwi1* and *qHMS4* further contribute to hybrid weakness and pollen sterility (Chen et al. 2013; Wang et al. 2022b). These findings provide a detailed genetic framework for understanding post-zygotic barriers.

In maize, unilateral CI represents a well-characterized pre-zygotic barrier controlled by loci such as *Ga1, Ga2,* and *Tcb1* (Wang et al. 2022a). These loci encode determinants that regulate pollen–silk interactions, with pectin methylesterases playing a central role in pollen tube growth and rejection (Chen et al. 2022). Similarly, in tomato, unilateral incompatibility is governed by *HT* family genes and additional loci such as *ui1.1* and *ui6.1*, which encode components of pollen recognition pathways (Covey et al. 2010; Li and Chetelat 2015). In wheat, crossability genes such as *Kr1* and *Kr2* restrict hybridization with related species (Alfares et al. 2009), while in *Brassica*, the S-locus genes (*SLG, SRK,* and *SCR/SP11*) regulate SI through ligand–receptor interactions (Okamoto et al. 2007). These studies reveal that CI is mediated by diverse molecular mechanisms involving signaling, cell wall modification, and reproductive development.

The emergence of CRISPR–Cas9 genome editing has transformed the ability to manipulate CI-associated genes. Targeted editing of incompatibility loci has enabled the creation of compatible hybrids in several crops. In maize, knockout of *ZmGa1F* removed female incompatibility barriers, allowing successful crosses (Zhang et al. 2023). Similarly, editing of S-RNase and *HT* genes in potato has generated self-compatible lines with improved seed production (Lee et al. 2023). CRISPR has also been used to overcome SI in rapeseed and tomato, demonstrating its broad applicability (Abhinandan et al. 2023). These advances highlight the potential of genome editing to bypass reproductive barriers and accelerate breeding. Despite these successes, challenges remain, including off-target effects, regulatory constraints, and stability of edited alleles across generations. Emerging tools such as base editing and prime editing offer improved precision and reduced genomic disruption, expanding the toolkit for overcoming CI.

While single-reference genomes have provided foundational insights into crop genetics, they are insufficient to capture the full extent of intraspecific diversity. Pan-genomes, representing the complete set of genes across multiple accessions, offer a more comprehensive view of genomic variation (Jha et al. 2022; Li et al. 2022). These resources are particularly valuable for identifying structural variations (SVs), TEs, and presence–absence variation that contribute to reproductive barriers (Bozan et al. 2023; Naik et al. 2026). Super pan-genomes further extend this concept by capturing the entire genetic repertoire of a species (Khan et al. 2020). However, their application to CI remains relatively underexplored. Recent studies demonstrate the potential of pan-genomes in addressing CI. In *Brassica*, pan-genomic analyses have identified genes associated with self- and CI, enabling targeted breeding strategies (Golicz et al. 2020). In rice, pan-genomic data have facilitated the identification of hybrid sterility genes and informed genome editing approaches to overcome these barriers (Zhou et al. 2023). In maize, pan-genome analyses have revealed allelic variation at the *Ga1* locus, enabling crosses between previously incompatible lines (Zhang et al. 2022). Similarly, in wheat, pan-genomes have improved understanding of hybrid incompatibility with CWR such as rye (Alfares et al. 2009).

Recent genomic strategies now provide a more realistic framework to dissect CI than single-reference mapping alone. Graph-based pan-genomes can represent alternate haplotypes, structural variations, copy-number changes, transposable element insertions, and presence–absence variation that are missed when diverse germplasm is aligned to one linear genome (Thudi et al. 2025). This is particularly relevant for CI because many reproductive barriers are controlled by complex loci, tightly linked gene clusters, duplicated genes, or rapidly evolving pollen–pistil recognition factors. In maize, the *Ga1/Ga2/Tcb1* systems already illustrate how compatibility can be governed by clustered determinants involved in pollen tube growth and silk-mediated rejection. Similar graph-enabled analyses should now be applied across CI systems to identify hidden SVs around S-loci, pollen-expressed genes, stylar factors, small RNA loci, and endosperm-regulatory regions. Recent pan-genome studies in barley, tomato, cucumber and potato demonstrate that CWR retain substantial non-reference sequence and SV diversity, providing a strong rationale to build CI-focused pan-genomes using compatible and incompatible parents, wild donors, bridge species and derived hybrids (Li et al. 2023; Jayakodi et al. 2024; Cheng et al. 2025). Such resources will allow breeders to move from marker discovery to “compatibility haplotype mining”, where favorable allelic combinations supporting pollen germination, pollen tube progression, fertilization and embryo survival can be selected before making difficult wide crosses.

Another important opportunity is to integrate pan-genomes with multi-omics and functional genomics. CI is not controlled only by coding variation; it also involves allele dosage, promoter divergence, TE-mediated regulation, chromatin state, small RNAs, imprinting and endosperm balance. Therefore, long-read assemblies, Hi-C based scaffolding, pan-genome graphs, single-cell transcriptomics of stigma/style tissues, pollen RNA profiling, methylome data and embryo/endosperm transcriptomes should be combined to resolve the timing and tissue specificity of incompatibility responses. Supporting this approach, *IbEXO70-26* has been identified as a strong candidate regulating CI in sweet potato due to its tissue-specific expression (Zhang et al. 2026), while in peach, reduced expression of *PpSLFL3* is associated with pollen tube arrest during cross-pollination (Wang et al. 2025). In practical breeding, this would mean comparing reciprocal crosses at defined stages—pollen hydration, tube emergence, stylar arrest, fertilization and early seed development—to identify whether failure is caused by pollen-side alleles, pistil-side rejection, dosage imbalance or post-zygotic lethality. Once candidate determinants are identified, CRISPR/Cas knockouts, base editing, promoter editing or allele replacement can be used to validate their role and create compatibility-neutral parents. This approach is more powerful than simply cataloging older sterility loci because it can reveal causal haplotypes and regulatory modules that determine whether wild alleles can enter the cultivated background.

Machine learning also offers a forward-looking strategy for predicting hybridization success before large crossing programs are attempted. Historical crossing records, parental genomic distance, graph-derived SV burden, k-mer profiles, S-locus haplotypes, pollen viability, flowering synchrony, ploidy level, endosperm genome dosage, and early embryo rescue outcomes can be used as predictors in supervised models. Random forests, gradient boosting, genomic best linear unbiased prediction, deep learning, and ensemble models can be trained to classify crosses as compatible, partially compatible, or highly incompatible. These models may also rank bridge parents and suggest reciprocal crossing direction where maternal effects are important. Recent reviews on genomic prediction and machine learning in plant breeding show that ML is increasingly useful when genomic, phenotypic, and environmental datasets are integrated, and the same principle can be extended to CI biology (Yoosefzadeh Najafabadi et al. 2023; Crossa et al. 2025). In future breeding pipelines, compatibility prediction should be treated as a pre-breeding decision-support tool, not merely as a post-crossing observation.

Together, graph pan-genomes, compatibility haplotypes, functional omics, genome editing, and ML-based cross prediction can transform CI research from descriptive genetics into predictive reproductive engineering. For crop improvement, the immediate priority should be to develop crop-specific CI panels consisting of compatible, incompatible and bridge-cross parents; phenotype them for pollen–pistil and embryo traits; sequence them using long-read technologies; and construct graph pan-genomes that capture both cultivated and wild diversity. These resources would help identify favorable compatibility alleles, avoid unproductive crosses, design better introgression schemes, and accelerate transfer of climate-resilience genes from secondary and tertiary gene pools. Thus, future CI research should not only ask which locus causes sterility or pollen rejection, but also which genomic architecture permits controlled gene flow across species boundaries.

## Synthetic biology and precision reproductive engineering

A promising new frontier is the application of synthetic biology to reprogram reproductive barriers, transforming CI from a fixed constraint into a programmable trait. Rather than bypassing incompatibility through traditional hybridization or embryo rescue, this approach seeks to design novel pollen–pistil recognition systems using modular synthetic components. Synthetic receptor–ligand modules, for instance, could be deployed to override endogenous S-locus signaling, enabling precise control over SI and interspecific compatibility (Liu and Stewart 2015). Furthermore, the development of toggleable SI/CI systems—whereby synthetic promoters, inducible transcription factors, or CRISPR-controlled effectors temporally modulate pollen rejection pathways—opens new avenues for cross-ploidy and cross-genus breeding. These innovations also allow for the strategic containment of transgenes using biological incompatibility switches, thus limiting gene flow in open environments and enhancing biosafety in genetically engineered crops (Burt and Crisanti 2018). In parallel, synthetic engineering of pollen–stigma compatibility interfaces, through domain-specific peptide mimics or apoplastic signaling reprogramming, holds promise for customizing inter-line or interspecies fertilization preferences. These advances blur the boundary between reproductive isolation and reproductive control, repositioning CI not merely as a barrier to be broken but as a designable feature for next-generation crop improvement. As synthetic biology platforms mature, they offer a transformative opportunity to rewire plant reproduction with precision, predictability, and ecological responsibility.

## Ecological and AI-assisted precision pollination: a non-genetic frontier for overcoming incompatibility

CI continues to constrain gene flow between crops and their CWR, but emerging non-genetic strategies at the interface of robotics, artificial intelligence (AI), and ecology offer alternative routes to bypass these barriers. Autonomous systems such as BrambleBee use LIDAR-guided navigation and computer vision to achieve precise floral targeting and pollen delivery, mimicking natural pollinators with high reliability (Ohi et al. 2018). Field-deployable robotic platforms have similarly demonstrated high flower coverage in crops, highlighting their potential as programmable agents for controlled crosses (Sapkota et al. 2023). Ecological approaches further complement these technologies. Specialist pollinators, including *Osmia spp*., enhance pollen transfer in crops where conventional vectors are inefficient, while increased pollinator diversity has been shown to reduce unilateral incompatibility through non-random pollen movement (Ratnayake et al. 2022). Advances in computer vision now allow real-time monitoring of floral receptivity and pollinator activity, enabling temporal precision in hybridization (Ratnayake et al. 2022). In parallel, machine learning models improve prediction of cross-compatibility and pollen performance.

## Future perspectives and conclusion

Despite substantial progress in identifying loci controlling CI, most studies remain restricted to a limited number of model crops. Functional validation of candidate genes across diverse taxa, integration of multi-omics datasets, and field validation of genomic predictions remain limited. Consequently, the transferability of current knowledge across crop species requires further investigation. Future progress in plant breeding will depend on resolving CI to enable efficient use of CWR. Recent advances in pan-genomics and genome editing provide a mechanistic entry point into these long-standing barriers. Pan-genomes, by capturing structural and presence–absence variation, are beginning to resolve loci underlying incompatibility in crops such as maize, rice, and *Brassica*, while integration with GWAS refines their functional interpretation. In parallel, CRISPR–Cas systems now permit targeted modification of incompatibility genes, offering a direct route to engineer hybrid compatibility and facilitate introgression from wild and tertiary gene pools. Expanding pan-genome resources across diverse crops, coupled with functional validation, will be critical to move from gene discovery to deployment. Integration with transcriptomics and predictive models may further enable compatibility forecasting, accelerating breeding pipelines. However, engineering compatibility also raises ecological considerations, particularly the potential for unintended gene flow into wild populations (Ellstrand et al. 2013). Despite these constraints, overcoming CI remains central to accessing resilience traits—including drought tolerance, salinity adaptation, and pest resistance—that are often confined to wild germplasm. Strategic integration of genomic and editing approaches thus offers a realistic pathway to broaden genetic diversity and enhance crop adaptation under climate change (Zsögön et al. 2022).

## Data Availability

Data availability does not apply to this article as no new data were created or analyzed in this study.
